# The Hidden Architects: Nitric Oxide and Redox Dynamics in Plant Stem Cell Homeostasis

**DOI:** 10.1002/bies.70048

**Published:** 2025-08-01

**Authors:** Jian Zeng, Xin'Ai Zhao, Jan U. Lohmann

**Affiliations:** ^1^ Department of Stem Cell Biology Centre For Organismal Studies Heidelberg University Heidelberg Germany

**Keywords:** environmental stress, epigenetics, hormone, meristem, nitric oxide, redox, ROS, stem cells

## Abstract

Plant stem cell homeostasis is a tightly controlled process governed by a complex network of transcription factors, hormones, signaling molecules, and various environmental factors. Among these, nitric oxide (NO) and redox signaling have emerged as critical regulators. This review examines the multifaceted role of NO in maintaining plant stem cell homeostasis, focusing on its influence through redox dynamics, DNA methylation, and hormonal regulation. We also explore the intricate cross‐talk between NO signaling and other key pathways, including environmental stimuli and the target of rapamycin (TOR) pathway, in balancing stem cell maintenance and differentiation within both shoot and root meristems. Additionally, we discuss NO's involvement in post‐translational modifications and transcriptional regulation, offering insights into its broader role in plant growth and development.

## Graphical abstract

This review highlights the dynamic interplay between nitric oxide (NO) and reactive oxygen species (ROS) in regulating plant stem cell renewal and differentiation. NO and ROS integrate intrinsic factors like epigenetic and hormonal signals with extrinsic cues, including environmental stress and nutrient‐TOR signaling, to maintain meristem function and developmental plasticity.

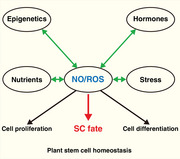

## 1 Introduction

**FIGURE 1:**
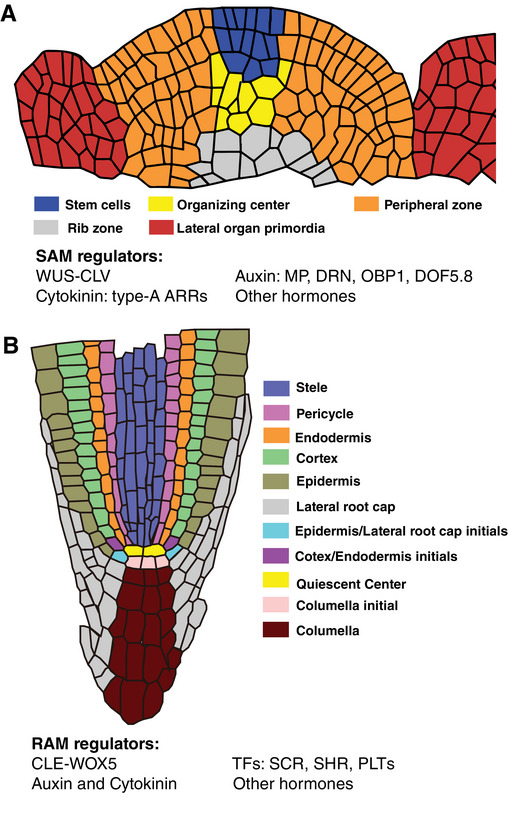
The shoot apical meristem (SAM) and root apical meristem (RAM). Schematic representation of the SAM at the shoot tip (A) and the RAM at the root tip (B) of *Arabidopsis*.

In plants, stem cells, located within the meristems, including the shoot apical meristem (SAM) and root apical meristem (RAM), are responsible for generating new organs and driving continuous growth throughout post‐embryonic development. Hence, SAM and RAM are key regions for maintaining the plant's ability to develop new tissues and adapt to environmental changes. In the SAM, cells are organized into distinct functional zones, including stem cells and their niche, the organizing center (OC), peripheral zone (PZ), rib zone, and organ primordia. Each of these zones plays a unique role in maintaining overall shoot meristem function and identity (Figure 1) [1, 2]. A key mechanism in SAM maintenance is the *WUSCHEL* (*WUS*)—*CLAVATA3* (*CLV3*) canonical feedback loop. WUS protein, produced in the OC, migrates into the overlying stem cells via cytoplasmic bridges called plasmodesmata to maintain their stemness. Conversely, CLV3, a small peptide secreted by the stem cells, represses *WUS* expression, ensuring the proper balance between stem cell maintenance and differentiation [3, 4, 5, 6]. In the RAM, stem cell identity and activity are regulated by the quiescent center (QC) and the adjacent stem cells, also called initials [7] (Figure 1). Transcription factors such as *SCARECROW* (*SCR*), *SHORT‐ROOT* (*SHR*), and *PLETHORAs* (*PLTs*), along with signaling pathways like the *CLAVATA3/ESR‐RELATED* (*CLE*)‐*WOX5* feedback module, play a key role in stem cell maintenance and balancing cell proliferation with differentiation [8, 9, 10, 11, 12]. Thus, these regulatory networks ensure proper plant growth and development by controlling the activity of stem cells.

Plant hormones are crucial regulators of stem cell homeostasis in both the SAM and RAM. Among these, auxin and cytokinin are widely recognized as central players in maintaining the balance between stem cell proliferation and differentiation. In the SAM, auxin induces differentiation, but also affects stem cell maintenance through multiple regulators such as *MONOPTEROS* (*MP*), *DORNROSCHEN* (*DRN*), or *DOF3.4/OBP1*, and *DOF5.8* [13, 14, 15]. Conversely, cytokinin promotes cell division via *MYB3R4*, as well as by stimulating *WUS* expression [16, 17]. Importantly, auxin and cytokinin activities are connected by direct crosstalk via type‐A *RESPONSE REGULATORs* (*ARR*), which are negative feedback regulators of cytokinin signaling [13]. On top of this cross‐talk, WUS directly controls both cytokinin and auxin signaling to bring about the appropriate balance for long‐term stem cell maintenance [18, 19, 20, 21].

In the RAM, auxin and cytokinin swap roles, and cytokinin plays an inhibitory role in cell division by activating type‐B *ARR* genes. The repression of cytokinin signaling through type‐A *ARR* is essential for defining root stem cells. Auxin, on the other hand, serves as a critical regulator in orchestrating both the maintenance and differentiation of root stem cells [22, 23, 24]. Beyond auxin and cytokinin, other hormones such as ethylene, gibberellins, and abscisic acid also contribute significantly to the regulation of stem cell activity [25, 26, 27, 28, 29, 30, 31, 32, 33, 34].

Nitric oxide (NO) and redox dynamics have emerged as important regulators of stem cell homeostasis in plant meristems. Originally studied for its role in animal systems, NO is now recognized as a key component in plant growth and development, influencing redox states, epigenetic modifications, and hormonal signaling pathways [35, 36, 37, 38, 39, 40, 41, 42]. NO interacts with auxin, cytokinin, gibberellin, and abscisic acid, forming a complex signaling network that integrates both developmental and environmental cues to regulate meristem homeostasis [43, 44, 45, 46, 47, 48, 49, 50]. Additionally, NO plays a significant role in plant stress responses, helping plants adapt to changing environmental conditions by influencing redox balance and gene expression [51, 52, 53, 54, 55, 56, 57, 58]. In this review, we focus on the role of NO in maintaining stem cell homeostasis within the SAM and RAM. We discuss how NO affects epigenetic modifications, particularly DNA methylation, and its interactions with the target of rapamycin (TOR) signaling pathway and stress response mechanisms. Furthermore, we examine NO's involvement in hormonal cross‐talk, highlighting its multifaceted influence on stem cell regulation.

## 2 Nitric Oxide and Redox Regulation of Plant Meristem Activity

**FIGURE 2:**
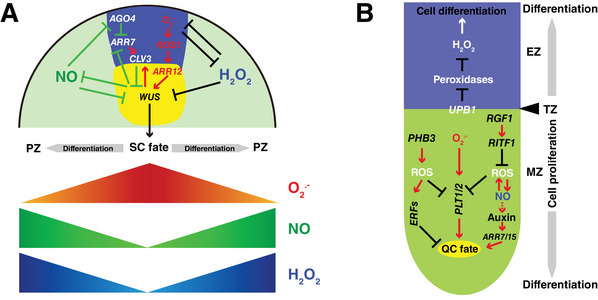
Nitric oxide (NO) and reactive oxygen species (ROS) signals in plant meristems. (A) NO and ROS regulate shoot apical meristem (SAM) activity through intricate signaling networks. NO accumulates in the peripheral zone (PZ), where it plays a crucial role in SAM regulation by restricting the expression of *WUSCHEL* (*WUS*) and promoting PZ cell fate. The role of NO in epigenetic regulation is highlighted by its control of ARGONAUTE 4 (AGO4) activity via S‐nitrosylation, which affects DNA methylation and contributes to SAM homeostasis. Superoxide (O_2_^.−^) also affects DNA methylation by activating the DNA demethylase REPRESSOR OF SILENCING 1 (ROS1), which regulates key SAM transcription factors such as *WUS* and *ARR12*. Maintaining a proper balance between different ROS species is crucial for SAM activity. (B) In the root apical meristem (RAM), ROS and NO work together to balance stem cell proliferation and differentiation. The transcription factor *UPBEAT1* (*UPB1*) regulates ROS homeostasis by repressing peroxidases such as *PRX39*, *PRX40*, and *PRX57*, thereby modulating the O_2_^.−^/H_2_O_2_ balance at the transition zone between the meristematic and elongation regions. This balance is critical for maintaining RAM size and activity. The mitochondrial protein PROHIBITIN3 (PHB3) ensures ROS balance and stem cell maintenance by regulating transcription factors like *WOX5* and *PLT1/PLT2*. Additionally, peptides such as RGF1 modulate ROS levels and RAM size through the RGF1‐inducible transcription factor 1 (RITF1).

Reactive nitrogen species (RNS), including NO, and reactive oxygen species (ROS), including the superoxide anion (O_2_^.−^) and hydrogen peroxide (H_2_O_2_), act as important signaling molecules in both developmental processes and stress responses across multicellular organisms [38, 39, 42, 54]. Superoxide is primarily produced by NADH dehydrogenase (Complex I) in mitochondria during cellular respiration and by NADPH oxidase in the plasma membrane. In addition, chloroplasts are also a major source of ROS, especially under high light or stress conditions, where over‐reduction of the electron transport chain leads to ROS production [59, 60]. Superoxide is scavenged by superoxide dismutase (SOD), which converts it into hydrogen peroxide (H_2_O_2_). In addition to SODs, hydrogen peroxide is generated directly by peroxidases and plays a role in signaling and defense. It is scavenged by enzymes like catalase and glutathione peroxidase [61, 62, 63, 64, 65]. NO is produced by NO synthase (NOS) in animals, which converts L‐arginine to NO, and by NOS‐like enzymes and nitrate reductase (NR) in plants, which reduce nitrate to nitrite and then to NO. NO is scavenged by antioxidants like glutathione and hemoglobin [66, 67, 68, 69]. Several studies have shown that these molecules not only participate in general developmental regulation but also interact with various genetic and epigenetic pathways to maintain meristem function. Among the factors involved in this regulatory network, *UPBEAT1 (UPB1)*, a basic helix‐loop‐helix (bHLH) transcription factor, has emerged as a significant player, orchestrating ROS homeostasis and regulating meristematic activity in both SAM and RAM (Figure 2). *UPB1* is crucial for balancing cell proliferation and differentiation in the RAM by repressing peroxidases activity (*PRX39*, *PRX40*, and *PRX57*), which alters the O_2_^.−^/H_2_O_2_ balance and negatively affects RAM size at the transition zone between the meristematic and elongation regions [70] (Figure 2). Similarly, in the SAM, *UPB1* regulates peroxidase expression. Loss of *UPB1* function results in increased peroxidase expression and reduced H_2_O_2_ levels, leading to a significant reduction in SAM size due to decreased differentiation of cells in the PZ. In contrast, overexpression of *UPB1* elevates H_2_O_2_ levels and increases SAM size without altering the stem cell domain [71], highlighting its effect on the PZ. Another key player is *PROHIBITIN3* (*PHB3*), a mitochondrial membrane protein, which is essential for ROS homeostasis in the RAM and contributes to stem cell maintenance. In *phb3* mutants, elevated mitochondrial ROS production, especially superoxide, disrupts quiescence and stem cell identity by affecting key transcription factors such as *WOX5* and *PLT1* (Figure 2). Notably, chemical scavenging of O_2_^.‐^ partially ameliorates these phenotypes, highlighting the critical role of redox balance in preserving root stem cell function [36, 72, 73]. Moreover, *PHB3* is important for facilitating NO production during H_2_O_2_‐induced stress, indicating potential cross‐talk between ROS and NO signaling in meristem regulation [36].

A fine‐tuned balance between O_2_^.−^ and H_2_O_2_ is essential not only for maintaining stemness in the RAM but also for determining shoot stem cell fate. In the SAM, elevated levels of O_2_^.−^ in stem cells promote *WUS* expression, while the accumulation of H_2_O_2_ in the PZ facilitates stem cell differentiation, with this balance regulated by the repression of SOD in stem cells and the activation of peroxidases [71]. H_2_O_2_ modulates O_2_^.−^ levels by repressing the expression of key oxidases, while low H_2_O_2_ content in stem cells fine‐tunes O_2_^.−^ levels to prevent excessive accumulation. This delicate balance between high O_2_^.−^ and low H_2_O_2_ is crucial for controlling stem cell number, as alterations in their ratio can promote differentiation and shift the boundary between stem and non‐stem cells [71] (Figure 2). Redox state is also connected to canonical cell to cell signaling: In the RAM, the application of the RGF1 peptide enhances RAM size and modifies the O_2_^.−^/H_2_O_2_ balance, with *RGF1‐inducible transcription factor 1* (*RITF1*) recognized as a key mediator. The phenotypes of *rgf* and *ritf* mutants, which exhibit reduced RAM size and altered PLT protein stability, further emphasize the vital role of redox regulation downstream of RGF peptides. Taken together, these findings highlight the importance of RGF1 and RITF1 in controlling root meristem development through their impact on ROS levels and stem cell maintenance [74] (Figure 2). A recent study reveals that cells of the RAM can perceive light independently of aboveground organs, where the light‐regulated transcription factor *ELONGATED HYPOCOTYL5* (*HY5*) directly activates *PER6*, which encodes a peroxidase protein, to eliminate H_2_O_2_ on the one hand, while repressing the know inhibitor of peroxidase *UPB1* on the other hand, thereby affecting ROS balance in the root to control root meristem activity [75]. These findings highlight the pivotal role of redox signaling in plant stem cell regulation, suggesting that precise manipulation of O_2_^.−^/H_2_O_2_ ratios could be a potential strategy for enhancing meristem function and development in both root and shoot meristems.

NO was initially considered as a metabolic byproduct, but early studies revealed its role in stress responses and signaling, raising questions about its function in plants versus animals. In the meantime, NO involvement in growth, stress tolerance, and development has been described, though the underlying effector mechanisms remain mostly elusive [66, 68, 76]. While the role of ROS in meristem regulation has been extensively studied, the role of NO is gaining attention only more recently. NO is recognized as a signaling molecule that interacts with ROS to influence redox dynamics, thereby modulating various stress responses and regulating meristem function. For example, NO plays a crucial role in cytokinin‐induced activation of *CYCD3;1* during cell proliferation, and overexpressing *CYCD3;1* can restore the meristematic defects observed in the RAM of the *noa1* mutant [43]. Moreover, the findings that the NO‐deficient *noa1* and *nia1/nia2* mutants exhibit small root meristems with abnormal cell divisions, along with reduced expression level of *WOX5* in *nia1/nia2* mutants, support the idea that NO signaling is important for controlling stem cell functions in root meristem [43, 44]. Similarly, in the SAM, the NO‐deficient mutants including *noa1* single mutants and *nia1/nia2/noa1* triple mutants show delayed leaf development and fewer floral buds, with smaller SAMs, increased *WUS* and *CLV3* expression, and reduced cell numbers in PZ, indicating NO's role in promoting PZ cell fate by restricting stem cell fate [41]. Consistent with these genetic findings, pharmacological perturbations revealed that NO limits *WUS* expression, with NO donors reducing *WUS* levels and NO scavengers increasing them. Notably, expression of the NO biosynthesis gene *NOA1* in stem cells reduces both *WUS* and *CLV3* levels, resulting in an expanded PZ and increased cell proliferation. This confirms that NO promotes PZ identity by restricting *WUS* expression [41] (Figure 2). Moreover, Shahid et al. (2019) identified around 20 differentially expressed genes (DEGs) related to stem cell function that respond to treatment with a NO donor, with *CLE12* showing the strongest positive correlation, revealing gene functions related to signal transduction and receptor activity. *clv1* mutants exhibit enhanced growth and pathogen resistance under NO‐mediated stress, and promoter analyses confirmed its activity in growth and stress regulation [77]. While the roles of ROS and NO have begun to emerge, direct evidence of the co‐regulation of meristem function by these two molecules remains limited. Hence, further work is required to elucidate the molecular mechanisms through which these signaling molecules interact to maintain stem cell homeostasis and regulate developmental processes in both the SAM and RAM.

## 3 Nitric Oxide‐Mediated DNA Methylation and Epigenetic Control of Plant Meristem Homeostasis

Unlike animals, where body plans are established early, most plant organs develop post‐embryonically from stem cells within the SAM and RAM, allowing plants to adapt their growth in response to environmental changes. Various factors are known to contribute to maintaining stem cell homeostasis in plant meristems, including NO, ROS, redox signaling, hormones and transcription factors. In addition to those regulators, epigenetic pathways involving DNA methylation, small RNA pathways, histone methylation, and histone acetylation also play a crucial role in meristem maintenance [41, 78, 79, 80, 81, 82, 83, 84]. For example, the loss of function in key epigenetic genes, such as *METHYLTRANSFERASE1* (*MET1*), *KRYPTONITE* (*KYP*), *JMJ14*, and *HAC1*, results in altered *WUS* expression and changes in the developmental rates of regenerated shoots in vitro, suggesting an epigenetic role in maintaining shoot meristem homeostasis and regeneration [78]. Genome‐wide analysis of DNA methylation in rice SAMs during vegetative and reproductive stages reveals that methylation at CHH sites is high in vegetative SAMs, particularly at transposable elements (TEs), but further increases in reproductive SAMs through the RNA‐dependent DNA methylation (RdDM) pathway. This indicates that significant changes in DNA methylation occur in the SAM before germ cell differentiation, likely serving as a protective mechanism against harmful TEs [82]. Similarly, in *Arabidopsis* SAMs, CHG methylation at TEs steadily increases, while CHH methylation decreases during the transition from the vegetative to the reproductive stage [81].

Despite significant progress in understanding NO and ROS signaling pathways, as well as the independent role of epigenetic regulation in plant stem cell maintenance, the connections between these systems remain largely unexplored. Emerging evidence suggests that interactions between NO, ROS signaling, and epigenetic pathways significantly contribute to the regulation of shoot meristem homeostasis [41, 84]. One intriguing finding highlights the role of NO upstream of epigenetic mechanisms in *Arabidopsis* SAMs. NO controls the expression and activity of *ARGONAUTE* 4 (*AGO4*), a key component of the RdDM pathway, both transcriptionally and through post‐translational modifications via S‐nitrosylation, a major physiological effect of NO. Through these mechanisms, NO, produced at the periphery of the SAM, restricts *AGO4* accumulation to the meristem's center where it controls DNA methylation (Figure 2). This represents one mechanism of communication between transit amplifying cells at the periphery and central stem cells, through modulation of epigenetic modifications in stem cells [41]. In line with this observation, two studies using S‐nitrosoproteomic analyses identify AGO1 and AGO4 as endogenously S‐nitrosylated proteins [85, 86], providing a potential direct link between NO signaling and epigenetic pathways. Importantly, AGO4 and the stem cell regulator WUS directly interact in a NO‐dependent manner and both proteins converge on common target genes. One prominent example is *ARR7*, one of the key A‐type ARRs involved in SAM function and auxin‐cytokinin cross‐talk, whose expression needs to be repressed for proper meristem activity [13, 18, 41] (Figure 2). Taken together, these findings underscore the crucial role of NO in regulating plant stem cell homeostasis by controlling DNA methylation.

Another breakthrough in understanding the redox‐epigenetic network regulating meristem activity is the discovery of the superoxide‐dependent activation of the DNA demethylase *REPRESSOR OF SILENCING 1* (*ROS1*) [84]. Loss‐of‐function mutations in *ros1* result in reduced expression of key meristem regulators like *WUS* and *CLV3*, leading to a reduction in meristem size. Moreover, *ROS1* functions downstream of ROS signaling to influence the expression of other critical transcription factors, such as *ARR12*, a component of cytokinin signaling. *ARR12* regulates the balance between stem cell proliferation and differentiation by controlling *WUS* expression [84] (Figure 2). These studies establish a direct link between NO, redox signaling, and epigenetic pathways, highlighting their convergence in maintaining meristems' activity in plants.

## 4 Hormonal Cross‐Talk: NO as a Central Mediator in Stem Cell Regulation

**FIGURE 3:**
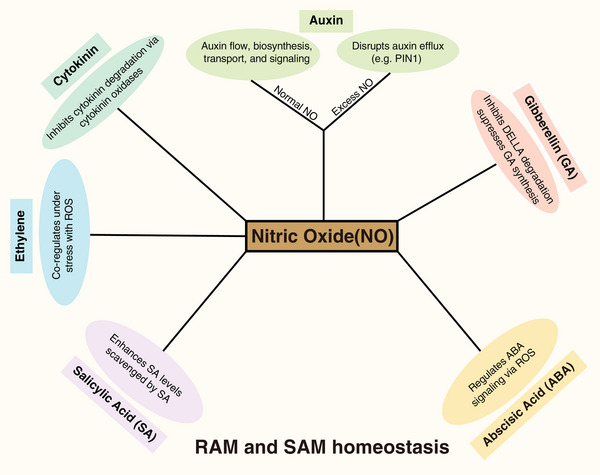
Nitric oxide (NO) signaling serves as a central hub integrating hormonal regulation of shoot apical meristem (SAM) and root apical meristem (RAM) homeostasis. NO maintains auxin flow and regulates auxin biosynthesis, transport, and signaling, while excess NO disrupts auxin efflux (e.g., PIN1). NO modulates cytokinin levels by inhibiting cytokinin degradation via cytokinin oxidases. NO and ethylene co‐regulate stem cell homeostasis under stress. Ethylene stabilizes ERFVIIs via PGB1, preventing oxidative damage mediated by NO. NO enhances SA levels to mitigate oxidative stress, SA scavenges NO, balancing its effects. NO modulates ABA homeostasis and post‐translationally regulates ABA signaling. ABA has dose‐dependent effects on RAM and SAM, influenced by ROS metabolism. NO inhibits GA signaling via S‐nitrosylation of DELLA proteins. NO suppresses GA synthesis (via GA3ox), negatively regulating root growth.

NO, in coordination with plant hormones such as auxin, cytokinin, ethylene, salicylic acid, and gibberellin, regulates stem cell division, differentiation, and homeostasis, thereby controlling overall plant growth. Auxin regulates stem cells through concentration gradients in plant meristems. In the RAM, high auxin levels promote stem cell activity, keeping them undifferentiated and supporting growth, with auxin transport as a key mechanism for developmental patterning. Auxin has been shown to interact with NO in maintaining stem cell niche homeostasis [44]. In *Arabidopsis* roots, NO accumulates in the cortex and endodermis stem cells as well as precursor cells. Mutants with impaired NO biosynthesis display smaller root meristems and irregular cell divisions. Furthermore, these mutants exhibit disruptions in auxin biosynthesis, transport, and signaling, suggesting that NO is essential in regulating stem cell fate and decision‐making processes. Conversely, excessive levels of NO reduce the activity of auxin efflux carrier *PIN‐FORMED 1* (*PIN1*) and impair the upward transport of auxin. The reduced auxin flow along with increased NO in turn disrupt cell division and growth at the root tip, leading to defects in the RAM [87]. These studies underscore the vital role of NO in fine‐tuning auxin dynamics to maintain stem cell populations in plant roots. Apart from the NO effect in roots, NO deficient mutants exhibit altered auxin maxima and mis‐regulated *PIN1* expression in the SAM, leading to changes in shoot architecture [88] (Figure 3).

NO also has been shown to interact with cytokinin signaling, the other canonical plant hormone involved in stem cell control [23, 89, 90]. NO modulates cytokinin levels by affecting cytokinin oxidases, which control cytokinin degradation. Elevated NO levels can reduce cytokinin degradation, maintaining a higher concentration of cytokinins and thus supporting stem cell proliferation [43, 91]. In *Arabidopsis*, mutations in *ALTERED MERISTEM PROGRAM1* (*AMP1*) cause enlarged SAMs and increased levels of endogenous cytokinin. *amp1* is also known as *cnu1* (continuous NO‐unstressed 1), and combining *cnu1* with the NO overproducing mutation *nox1* suppresses the phenotype, suggesting that it is indeed caused by reduced levels of NO. Consistently, treating *nox1* mutants with trans‐zeatin also rescues, implying that cytokinin likely inhibits NO activity in the SAM [92, 93] (Figure 3). In addition, NO serves as a key mediator in controlling the reprogramming of mitotic cycles by activating *CYCD3;1* in response to cytokinin during callus formation and meristem maintenance. Consequently, overexpression of *CYCD3;1* can compensate for the meristematic defects observed in the NO‐deficient *nos1/noa1* mutant in both SAM and RAM [43].

Emerging evidence suggests that NO and ethylene act together to regulate plant stem cell homeostasis, particularly in response to environmental stress [49, 55, 94, 95, 96]. During submergence, ethylene enhances the stability of group VII Ethylene Response Factor (ERFVII) by increasing the expression of *PHYTOGLOBIN1* (*PGB1*), a NO‐scavenger, which in turn prevents the overaccumulation of ROS and protects stem cells in the shoot and root meristems from oxidative damage [55]. In maize, phytoglobins protect the RAM from hypoxia‐induced programmed cell death (PCD). This process is initiated by NO and mediated by ethylene via ROS [97] (Figure 3).

Salicylic acid (SA) is known for its role in immune signaling, but SA can also modulate stem cell function by altering hormonal balances, such as auxin and cytokinin signaling. SA may also affect the redox balance in stem cells, impacting their ability to proliferate and maintain growth under stress, helping plants adapt to adverse conditions [98, 99, 100]. SA plays a key role in a stem cell‐specific antiviral defense mechanism in plants, activating plant‐encoded RNA‐dependent RNA polymerase. This enzyme enhances antiviral RNA interference (RNAi) in infected stem tissues, supplying stem cells with RNA‐based viral sequence information to inhibit viral replication and protect them from RNA virus infections [100]. NO and SA interact in both positive and negative ways to control these plant defense responses. NO signaling enhances SA levels, which aids in mitigating NO‐induced oxidative stress. In turn, SA acts as a scavenger for NO and its related molecules, balancing their effects [101, 102] (Figure 3). Given that NO controls shoot meristem activity via an epigenetic pathway and *WUS* has been shown to play a role in preventing virus accumulation in *Arabidopsis* shoot stem cells [41, 103], it would be interesting to investigate whether NO is also involved in SA‐mediated defense mechanisms against other pathogens.

Abscisic acid (ABA) can either stimulate or inhibit meristem activity in a dose‐dependent manner. Low concentrations of exogenous ABA have been shown to increase the size of RAM, while high concentrations inhibit it, with ROS mediating its inhibitory effect [29, 104, 105]. Interestingly, ABA concentrations that inhibit RAM activity increase meristem size in the shoot, indicating that the effects of ABA are influenced by tissue context. One example is the long‐distance transport of auxin from the shoot to the root, which enhances cell division in the RAM [106]. Additionally, NO metabolism involves various biochemical mechanisms that regulate ABA homeostasis, NO post‐translational modifications regulate ABA signaling in response to environmental factors that involve ROS metabolism [104]. However, the interactions between ABA and NO have been primarily studied in contexts such as seed dormancy, germination, stomatal movement, leaf senescence, fruit ripening, and stress responses, with little focus on stem cell homeostasis thus far.

The biological functions of gibberellins, which generally promote stem elongation and differentiation, are also fine‐tuned by NO [37, 107, 108, 109] (Figure 3). NO negatively affects gibberellin signaling via S‐nitrosylation of the main DELLA repressor RGA at Cys‐374, inhibiting its proteasomal degradation and thus leading to inhibition of root growth [47]. NO also inhibits primary root growth partially by repressing GA3ox‐catalyzed GA3 synthesis in *Arabidopsis* [109] (Figure 3). However, how NO and gibberellin converge on plant stem cell homeostasis requires further exploration.

## 5 Nitric Oxide and Stress Response in Plant Meristems

**FIGURE 4:**
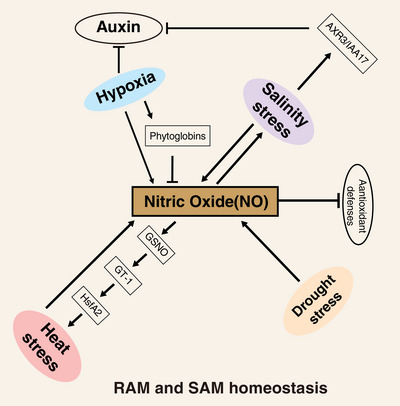
Nitric oxide (NO) signaling serves as a central hub integrating stress‐mediated regulation of shoot apical meristem (SAM) and root apical meristem (RAM) homeostasis. NO levels increase in the root tip under hypoxic conditions, depleting RAM by reducing auxin accumulation in WOX5‐expressing quiescent center (QC) cells. Phytoglobins mitigate this effect by scavenging NO and maintaining meristem function. Heat stress induces NO biosynthesis, which interacts with glutathione to form S‐nitrosoglutathione (GSNO). GSNO triggers systemic heat responses by S‐nitrosylating transcription factors like GT‐1, activating stress‐responsive genes (e.g., HsfA2) to protect meristematic cells. Salt stress elevates NO levels, stabilizing proteins (e.g., AXR3/IAA17) that repress auxin signaling, thereby reducing RAM size. NO synthesis is upregulated under drought, alleviating its effects by enhancing antioxidant defenses, modulating transcriptional programs, and maintaining cell homeostasis.

Plants must adapt to the specific environments they inhabit, making it essential for them to maintain stem cells under unfavorable or fluctuating conditions. NO biosynthesis is controlled by multiple hormonal and environmental stimuli to trigger protective responses and to modulate energy metabolism, which contribute to plant growth and development. During stresses like hypoxia, high temperature, or drought, NO interacts with other signaling molecules such as auxin and ROS to modulate stress responses. Phytoglobins, heme‐containing proteins that act as scavengers of NO, are expressed in the root tip [110, 111] and are induced by biotic and abiotic stresses (Figure 4). These proteins protect meristem function and prevent meristematic cell death under suboptimal environmental conditions [112, 113]. The primary auxin, indole‐3‐acetic acid (IAA), plays a crucial role in integrating environmental signals into the developmental growth responses of plants under stress [114, 115, 116]. In *Arabidopsis*, exposure to abiotic stress can inhibit root meristem growth by reducing auxin levels, a process that is regulated by NO [117]. The level of NO production varies within different parts of the root, with the highest production occurring near the root tip, which influences the activity of QC cells [118, 119]. During waterlogging, hypoxic conditions elevate NO levels in the root, leading to the depletion of the RAM due to reduced accumulation of auxin in *WOX5* expressing QC cells. Phytoglobins interfere with NO signaling, and overexpression of *PGB1* is sufficient to retain PIN‐mediated auxin maxima in the root tips, maintain *WOX5* expression in the QC, and preserve meristem function [120, 121] (Figure 4).

High temperature is among the biggest abiotic stress challenges for agriculture, causing irreversible damage. NO biosynthesis mutants such as *noa1* and *nia1/nia2*, exhibit increased sensitivity to heat stress. However, their thermotolerance can be improved through exogenous application of NO donors [122, 123]. Multiple studies suggest that NO plays a key role in mediating a range of plant responses during heat stress, including photosynthesis, oxidative defense, osmolyte accumulation, gene expression, and protein modifications, which was well documented by Parankusam [53]. It is well recognized that exposure to elevated temperatures generally results in a swift increase in NO production across various plant species [52, 53, 57, 123, 124, 125, 126, 127] (Figure 4). For example, heat stress triggers a significant surge in NO production in the inflorescence apex of *Arabidopsis*. In this context NO interacts with glutathione to form S‐nitrosoglutathione (GSNO), which can rapidly travel from the shoot to the root through the vascular system. GSNO acts as a signaling molecule that initiates heat stress responses throughout the plant by S‐nitrosylating the trihelix transcription factor GT‐1. This modification enhances the binding affinity of GT‐1 to NO‐responsive elements within the *HsfA2* promoter, thereby activating the expression of *HsfA2* and its downstream target genes, ultimately improving the plant's thermotolerance [57] (Figure 4).

Salinity stress is marked by a dual negative impact, leading to both cell dehydration and toxicity. These in turn adversely affect plant growth by disrupting water uptake, causing ion toxicity, and impairing photosynthesis, leading to stunted growth, reduced biomass, and compromised reproductive success [128] (Figure 4). Endogenous NO levels are increased under salt stress in various plant species. Here, NO mitigates oxidative damage, promotes ion homeostasis, enhances antioxidant enzyme activity, and modulates gene expression to improve plant tolerance to salt stress [128, 129, 130, 131, 132, 133, 134, 135, 136]. In *Arabidopsis*, salt stress reduces RAM size by reducing the expression of *PIN* genes, which results in lower auxin levels at the root tip. At the same time, it also promotes the stabilization of *AXR3/IAA17*, which represses auxin signaling. Additionally, salt stress increases NO accumulation, and blocking NO production reverses the effects of salt stress on roots [137].

Drought is among the most common and unpredictable environmental stressors that limit water availability, reduce cell expansion, photosynthesis, and nutrient uptake, leading to stunted growth and development, along with significant reductions in crop yield. NO is essential for enhancing drought tolerance, and various plant species have shown increased NO synthesis in response to water deficit [123]. NO alleviates drought stress at the morpho‐anatomical, physiological, and biochemical levels, helping plants adapt to water‐limited conditions [56] (Figure 4). Shahid et al. (2019) conducted a comprehensive analysis of NO‐induced genes associated with stem cell regulation in *Arabidopsis*, revealing that the promoter regions of these NO‐responsive genes contain cis‐elements that contribute to tolerance against both abiotic and biotic stresses, including ABRE (TACGTG), which is crucial for regulating responses to osmotic and drought stress [77]. However, how NO and drought stress affect plant stem cell homeostasis remains largely unexplored.

## 6 TOR Signaling and Nitric Oxide: Coordinating Stem Cell Regulation and Energy Balance

**FIGURE 5:**
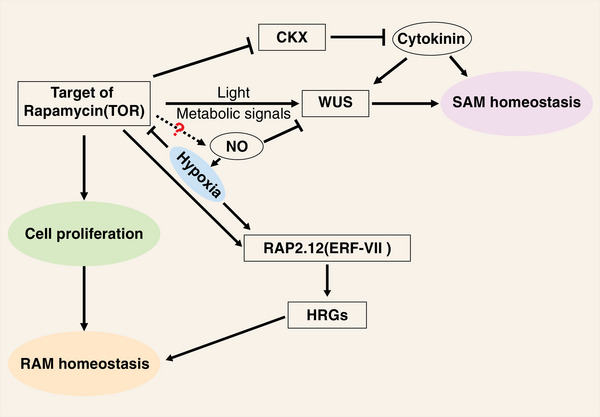
Regulation of shoot apical meristem (SAM) and root apical meristem (RAM) homeostasis by target of rapamycin (TOR) and nitric oxide (NO). TOR and NO act as pivotal regulators in the maintenance of SAM and RAM activity. TOR promotes *WUSCHEL* (WUS) expression in the central zone of the SAM by responding to light, metabolic signals. TOR negatively regulates cytokinin catabolism by inhibiting the translation of CKX (cytokinin oxidase/dehydrogenase), thereby maintaining higher cytokinin levels, which support stem cell proliferation. In RAM, TOR supports cell division and energy balance required for root development and growth. TOR regulates hypoxia responses via phosphorylation of group VII ethylene response factors (ERF‐VII, e.g., RAP2.12). This activation of hypoxia‐responsive genes (HRGs) ensures survival under low‐oxygen conditions. During hypoxia, low ATP levels reduce TOR activity, leading to decreased activation of ERF‐VII transcription factors and hypoxia‐responsive pathways. NO accumulates predominantly in the peripheral zone (PZ) of the SAM and represses central stem cell fate by inhibiting *WUS* expression; therefore, NO signalling contributes to fine‐tuning the balance between central zone and PZ activities in the SAM. It is unclear whether TOR directly phosphorylates WUS or if NO is involved in TOR‐mediated WUS regulation in the SAM.

TOR is a deeply conserved protein kinase that serves as a key signaling hub in diverse physiological and developmental pathways: it plays a role in regulating plant stem cell function, coordinating nutrient and energy signals, controlling the cell cycle, and modulating hormonal pathways in response to environmental stimuli [138]. The plant *TOR* gene is predominantly expressed in meristem regions characterized by high cell proliferation, which supports the idea that TOR is essential for regulation of the activation of both SAM and RAM [138, 139]. Pfeiffer et al. found that TOR activity is required for the expression of WUS in response to light and metabolic signals, and it turns out that TOR negatively regulates the translation of mRNAs encoding CKX cytokinin catabolic enzymes [139, 140] (Figure 5). TOR controls group VII ethylene response factors ERF‐VII by phosphorylating two serine residues at the C terminus of RAP2.12, a major ERF‐VII transcription factor. This phosphorylation by TOR is necessary for the full activation of RAP2.12's transcriptional activity under hypoxic conditions. When energy levels are low due to inefficient ATP production under hypoxia, TOR activity decreases, which in turn dampens the activation of ERF‐VII and reduces the induction of hypoxia‐responsive genes (HRGs). This mechanism also involves NO and ethylene, allowing plants to coordinate oxygen and energy sensing, modulating the hypoxia response based on energy availability, which is critical for survival during submergence [141] (Figure 5). NO accumulates to higher levels in the PZ compared to the central zone in the *Arabidopsis* SAM, and NO signaling represses central cell fate via repressing *WUS* expression [41]. However, it remains unclear whether TOR kinase directly phosphorylates WUS and if NO is involved in this process to regulate stem cell homeostasis.

## 7 Conclusions and Perspective

Future research on NO and ROS in relation to plant meristems offers promising avenues for advancing our understanding of plant growth and development. Both NO and ROS have been implicated in regulating stem cell activity, meristem maintenance, and hormonal signaling pathways, such as those involving auxin and cytokinin, however, the underlying molecular mechanisms remain largely unexplored. A critical step forward will be the development of new methods for more precisely detecting NO and ROS production within live plant cells, particularly its dynamic distribution in meristematic cells in response to external factors. Innovative technologies such as single‐cell multiomics and single‐cell spatial omics hold significant promise for identifying NO and ROS targets at both transcriptional and post‐transcriptional levels. These approaches will help uncover the precise molecular mechanisms by which NO and ROS interact with other signaling molecules, especially plant hormones, revealing critical insights into how plants balance growth and stress responses, especially in meristematic tissues.

Moreover, it remains unclear how NO and ROS interact with other environmental factors to coordinate meristem activity. How can these interactions be leveraged for crop improvement, and what are the specific upstream and downstream components involved in NO‐ and ROS‐mediated stem cell regulation? Furthermore, investigating the role of NO and ROS in modulating plant responses to abiotic stresses such as drought, salinity, and extreme temperatures, could pave the way for strategies to enhance crop resilience. How can NO and ROS signaling be precisely modulated to improve stress tolerance without compromising plant growth, and what are the trade‐offs associated with its targeted manipulation for enhancing crop yield in specific agricultural contexts?

In terms of agricultural applications, manipulating NO and ROS signaling pathways offers potential for improving crop performance, since by modulating NO and ROS levels, root and shoot meristem activity could be enhanced, resulting in optimized plant architecture, higher yields, and better stress adaptation.

In summary, research on NO and ROS provides important insights into the regulation of plant meristems and holds potential for informing breeding strategies aimed at improving developmental plasticity and stress resilience. Further investigation into their specific roles and molecular targets in meristem regulation will be crucial to translating these findings into practical applications in agriculture.

## Author Contributions

J.U.L, J.Z., and X.Z. conceived of and wrote the manuscript.

## Conflicts of Interest

The authors declare no conflicts of interest.

## Data Availability

Data sharing not applicable to this article as no datasets were generated during the current study.
